# Epidemiological characteristics, virulence genes, and antimicrobial resistance analysis of *Vibrio parahaemolyticus* in diarrheal cases in Huzhou City from 2021 to 2023

**DOI:** 10.3389/fmicb.2025.1551984

**Published:** 2025-08-29

**Authors:** Xiaofang Wu, Liping Chen, Xiaojuan Zhu, Lei Ji, Fenfen Dong

**Affiliations:** Huzhou Center for Disease Control and Prevention, Huzhou, China

**Keywords:** *V. parahaemolyticus*, virulence genes, antibiotic resistance, serovars, epidemiological features

## Abstract

*Vibrio parahaemolyticus* has emerged as a predominant cause of seafood-related infections globally. Despite this, a comprehensive analysis of its epidemiological traits and antimicrobial resistance patterns in Huzhou City remains lacking. Our study isolated 250 strains of *V. parahaemolyticus* from a total of 6,404 diarrhea patients sampled across six hospitals in Huzhou from 2021 to 2023. Epidemiological analysis revealed higher infection rates in warmer seasons, with the majority of cases occurring in individuals aged from 25 to 64. No significant gender-based difference was observed in the prevalence of *V. parahaemolyticus*. Serotype analysis identified O10:K4 as the predominant serotype. 93.20% (233/250) of clinical isolates harbored the *tdh* gene, while 4.0% (10/250) carrying the *trh*. Antimicrobial sensitivity testing indicated a strikingly high resistance rate of 95.56% (172/180) to cefazolin among clinical isolates. The cgMLST-based minimum spanning tree analysis revealed that the O4:KUT clinical isolates segregated into two distinct clusters, ST3 and ST2516, with considerable evolutionary divergence between them. In contrast, the O10:K4 and O3:K6 serotypes exhibited closer phylogenetic proximity. This study comprehensively characterizes *V. parahaemolyticus* in Huzhou, revealing critical insights into its epidemiology, virulence factors, and antibiotic resistance patterns, thereby enhancing our knowledge of its public health risks.

## 1 Introduction

*Vibrio parahaemolyticus* is a Gram-negative, spore-forming bacillus with halophilic properties (Preeprem et al., 2019), widely distributed in coastal areas, marine sediment, and seafood, and is prone to contaminate fish, shrimp, shellfish, and other aquatic products (Zhou et al., 2022). Consumption of undercooked or cross-contaminated food can lead to symptoms such as abdominal pain, diarrhea, acute gastroenteritis, and septicemia. *V. parahaemolyticus* is recognized as a major cause of foodborne disease outbreaks in many Asian countries (Letchumanan et al., 2014). In China, foodborne diseases are mainly caused by pathogenic microorganisms, with outbreaks primarily caused by *V. parahaemolyticus, Salmonella* and *Staphylococcus aureus* (Geng et al., 2019). *V. parahaemolyticus* is one of the most common causative agents of foodborne illness associated with ready-to-eat (RTE) foods (Xie et al., 2016). It is reported that in Zhejiang Province, pathogenic bacteria constitute the primary causative agents of foodborne disease outbreaks, with *V. parahaemolyticus* being the most prevalent species (Chen et al., 2023). Research indicates that *V parahaemolyticus* has emerged as the leading cause of foodborne diseases in Zhejiang Province, accounting for 58.4% of bacterial outbreaks (Chen et al., 2022). According to statistics, *V. parahaemolyticus* is also one of the main pathogens causing bacterial foodborne outbreaks and sporadic foodborne diarrhea cases in Huzhou City (Yan et al., 2024, 2020). This study selected 250 strains of *V. parahaemolyticus* isolated from sporadic diarrhea cases in at least, from six hospitals of Huzhou City from 2021 to 2023 and used real-time fluorescence PCR and microbroth dilution methods to detect virulence genes, serotypes, and drug sensitivity, in order to understand the epidemiological characteristics, presence of virulence genes, and the current status of antimicrobial resistance of *V. parahaemolyticus* in sporadic diarrhea cases in Huzhou City.

## 2 Materials and methods

### 2.1 Sample collection and *V. parahaemolyticus* serotyping

From January 2021 to December 2023, six hospitals including Wuxing District People's Hospital, Deqing County People's Hospital, Changxing County People's Hospital, Nanxun District People's Hospital, Anji County People's Hospital, and Huzhou First People's Hospital for foodborne disease surveillance in Huzhou City tested 6,404 diarrhea case specimens defined by having a daily bowel movement frequency of ≥3 times and abnormal stool characteristics (loose stools, watery stools, mucoid stools, or bloody stools, etc.) for *V. parahaemolyticus* (https://www.who.int/news-room/fact-sheets/detail/diarrhoeal-disease), and a total of 250 *V. parahaemolyticus* strains were isolated. These included 88 isolations from 2021, 86 isolations from 2022, and 76 isolations from 2023. The number of isolates from each hospital per year was detailed in Table 1. The isolation, identification and serotyping of *V. parahaemolyticus* were performed as previously described (Zhang et al., 2024a). The serotyping of *V. parahaemolyticus* isolates was conducted using standardized slide agglutination assays with commercially available antisera (Denka Seiken Ltd., Tokyo, Japan). The complete serotyping scheme included 11 O (somatic) and 65 K (capsular) antigenic determinants. Isolates were classified into serotypes based on their unique O:K antigen combinations. Isolates that failed to demonstrate K-antigen agglutination were classified as K-untypeable (KUT) serotypes (Jones et al., 2012).

**Table 1:** Annual case numbers and positive detection rates from six hospitals in Huzhou City (2021–2023).

**Hospitals**	**2021**		**2022**		**2023**	
	**Cases**	**Number/positive detection rate/%**	**Cases**	**Number/positive detection rate/%**	**Cases**	**Number/positive detection rate/%**
Huzhou First People's Hospital	482	8/1.66	594	16/2.69	403	5/1.24
Deqing County People's Hospital	197	14/7.11	546	23/4.21	334	15/4.50
Changxing County People's Hospital	244	19/7.79	617	19/3.08	487	32/6.57
Anji County People's Hospital	374	9/2.41	277	9/3.25	238	6/2.52
Wuxing District People's Hospital	387	34/8.79	264	10/3.79	231	6/2.60
Nanxun District People's Hospital	229	4/1.75	254	9/3.54	246	12/4.88
Summary	1,913	88/4.60	2,552	86/3.37	1,939	76/3.92

### 2.2 Statistical analysis of temporal distribution of cases

Strain distribution analysis was conducted in Excel according to specimen collection timelines, with data presentation optimized through bar chart visualization using KaleidaGraph 4.5. The tables were prepared using Microsoft Word.

### 2.3 Virulence gene detection

A fresh bacterial colony was suspended in 200 μL sterile water, heat-lysed at 100 °C for 10 min, and centrifuged at 10,000 × g for 5 min. The DNA-containing supernatant was collected and stored at −80 °C until further use (Yan et al., 2020). Pathogenic *V. parahaemolyticus* can produce either TDH, TRH, or both (Nishibuchi and Kaper, 1995). The gene *tlh* is widely considered to be a marker for *V. parahaemolyticus* (Bej et al., 1999; DePaola et al., 2003). The detection of *tlh/tdh/trh* virulence genes were performed according to the instructions of the *V. parahaemolyticus* (TLH, TDH, TRH) triplex real-time fluorescent PCR detection kit (Biogen Biological Co., Ltd., Shenzhen, China). The detailed method was performed as previously described (Zhang et al., 2024b). Quality control was performed using the manufacturer-provided negative and positive controls included in the kit to verify test accuracy.

### 2.4 Susceptibility test

In accordance with the Clinical and Laboratory Standard Institution guidelines (CLSI M100-S23) and ChinaPIN-2022-TYJS004, the microbroth dilution method was used to determine the minimal inhibitory concentration (MIC) of *V. parahaemolyticus* against antimicrobial agents. The 13 antimicrobial agents included penicillins: ampicillin (AMP) (64, 32, 16, 8, 4, 2, 1 μg/mL), ampicillin/sulbactam (AMS) (64/32, 32/16, 16/8, 8/4, 4/2, 2/1, 1/0.5 μg/mL); cephalosporins: cefotaxime (CTX) (16, 8, 4, 2, 1, 0.5, 0.25 μg/mL), ceftazidime (CAZ) (32, 16, 8, 4, 2, 1, 0.5 μg/mL), cefuroxime (CFX) (32, 16, 8, 4, 2, 1, 0.5 μg/mL), cefazolin (CFZ) (32, 16, 8, 4, 2, 1, 0.5, 0.25 μg/mL); carbapenems: imipenem (IPM) (8, 4, 2, 1, 0.5, 0.25 μg/mL); aminoglycosides: gentamicin (GEN) (32, 16, 8, 4, 2, 1 μg/mL); tetracyclines: tetracycline (TET) (32, 16, 8, 4, 2, 1 μg/mL); quinolones: nalidixic acid (NAL) (64, 32, 16, 8, 4, 2 μg/mL), ciprofloxacin (CIP) (32, 16, 8, 4, 2, 1, 0.5, 0.25, 0.12, 0.06, 0.03, 0.015 μg/mL); chloramphenicol: chloramphenicol (CHL) (64, 32, 16, 8, 4, 2 μg/mL); and sulfonamides: trimethoprim/sulfamethoxazole (SXT) (8/152, 4/76, 2/38, 1/19, 0.5/9.5, 0.25/4.75 μg/mL). The results were expressed as sensitive (S), intermediate (I), and resistant (R). *Escherichia coli* ATCC 25922 was included as a quality control strain.

### 2.5 Statistical analysis

Statistical processing was conducted using SPSS 23.0 software, and data analysis was performed with the chi-square test, with a difference considered statistically significant at *p* < 0.05.

### 2.6 Core genome multilocus sequence typing (cgMLST) profiling and minimum spanning tree-based phylogenetic analysis

We downloaded the 121 sequences of clinical isolates from 2021 to 2023, which were previously submitted to NCBI under BioProject accession numbers PRJNA1115946, PRJNA1072230, and PRJNA1071824 in our prior studies, for subsequent analysis. Detailed strain information was provided in Supplementary Table S1. For the characterization of *V. parahaemolyticus*, seven housekeeping genes (*dnaE, gyrB, recA, dtdS, pntA, pyrC*, and *tnaA*) were analyzed. A multilocus sequence typing (MLST) scheme was applied to the 121 genomes using mlst v2.23.0, generating an MLST profile for each isolate. The chewBBACA suite was employed for cgMLST analysis, beginning with schema construction using the CreateSchema module (Silva et al., 2018). Subsequently, allele identification was performed across 95% loci for each isolate through the AlleleCall module, producing a 95% allele profile matrix. To maintain analytical rigor, potential paralogous loci were systematically filtered using the RemoveParalogs module with default parameters, thereby eliminating sequences that could compromise cgMLST interpretation. The combination of alleles in each isolate formed an allelic profile that was used to generate minimum spanning trees (MSTs). The MSTs were constructed in BioNumerics using the cgMLST allele profiles as input data (Blanc et al., 2020). The unweighted pair group method with arithmetic mean (UPGMA) was used as the clustering algorithm. The pairwise distance was calculated by counting the number of pairwise allele differences.

## 3 Results

### 3.1 Basic case information

From 2021 to 2023, a total of 6,404 foodborne diarrhea case specimens were collected from six hospitals, yielding 250 isolates of *V. parahaemolyticus*, with an overall positive detection rate of 3.90%. Specifically, in 2021, 1,913 specimens were collected with a positive detection rate of 4.60%. The positive detection rates of Huzhou First People's Hospital, Deqing County People's Hospital, Changxing County People's Hospital, Anji County People's Hospital, Wuxing District People's Hospital, and Nanxun District People's Hospital were 1.66%, 7.11%, 7.79%, 2.41%, 8.79%, and 1.75%, respectively. In 2022, 2,552 specimens were collected with a rate of 3.37%. The positive detection rates for the six hospitals mentioned above were 2.69%, 4.21%, 3.08%, 3.25%, 3.79%, and 3.54%, respectively. In 2023, 1,939 specimens were collected with a rate of 3.92%. The six hospitals reported positive detection rates of 1.24%, 4.50%, 6.57%, 2.52%, 2.60%, and 4.88%, respectively (Table 1). The annual case numbers and positivity rates for the six specific hospitals from 2021 to 2023 are detailed in Table 1. The variation in the detection rate of *V. parahaemolyticus* across the different years was statistically significant (χ^2^= 4.413, *p* < 0.05).

### 3.2 Case population distribution

Among the 6,404 diarrhea infection cases, there were 3381 males and 3023 females.

Positive detection rates of *V. parahaemolyticus* in males and females were 3.67% and 4.17%, respectively, with no statistically significant difference between genders (χ^2^= 0.936, *p* > 0.05). The age distribution of infected cases ranged from 5 months to 97 years, with the highest infection rates in the 45–64 and 25–44 age groups at 6.74% and 4.34%, respectively, and no detection in the age group under 5 years (Table 2). The distribution of detection across different age groups was statistically significant (χ^2^= 59.822, *p* < 0.05).

**Table 2:** Detection of *V. parahaemolyticus* in Huzhou City from 2021 to 2023.

**Population Characteristics**	**2021**		**2022**		**2023**		**Summary**	
	**Number**	**Rate (%)**	**Number**	**Rate (%)**	**Number**	**Rate (%)**	**Number**	**Rate (/%)**
**Gender**								
Male	985	4.37	1,389	3.02	1,007	3.87	3,381	3.67
Female	928	4.85	1,163	3.78	932	3.97	3,023	4.17
**Age**								
< 5	141	0.00	229	0.00	182	0.00	552	0.00
5–14	79	1.27	121	1.65	117	0.85	317	1.26
15–24	339	1.77	410	2.44	317	4.42	1,066	2.81
25–44	788	5.71	993	3.12	797	4.52	2,578	4.34
45–64	369	6.78	486	7.41	362	5.80	1,217	6.74
≥65	197	5.58	313	2.24	164	2.44	674	3.26

### 3.3 Temporal distribution of cases

**Figure 1:**
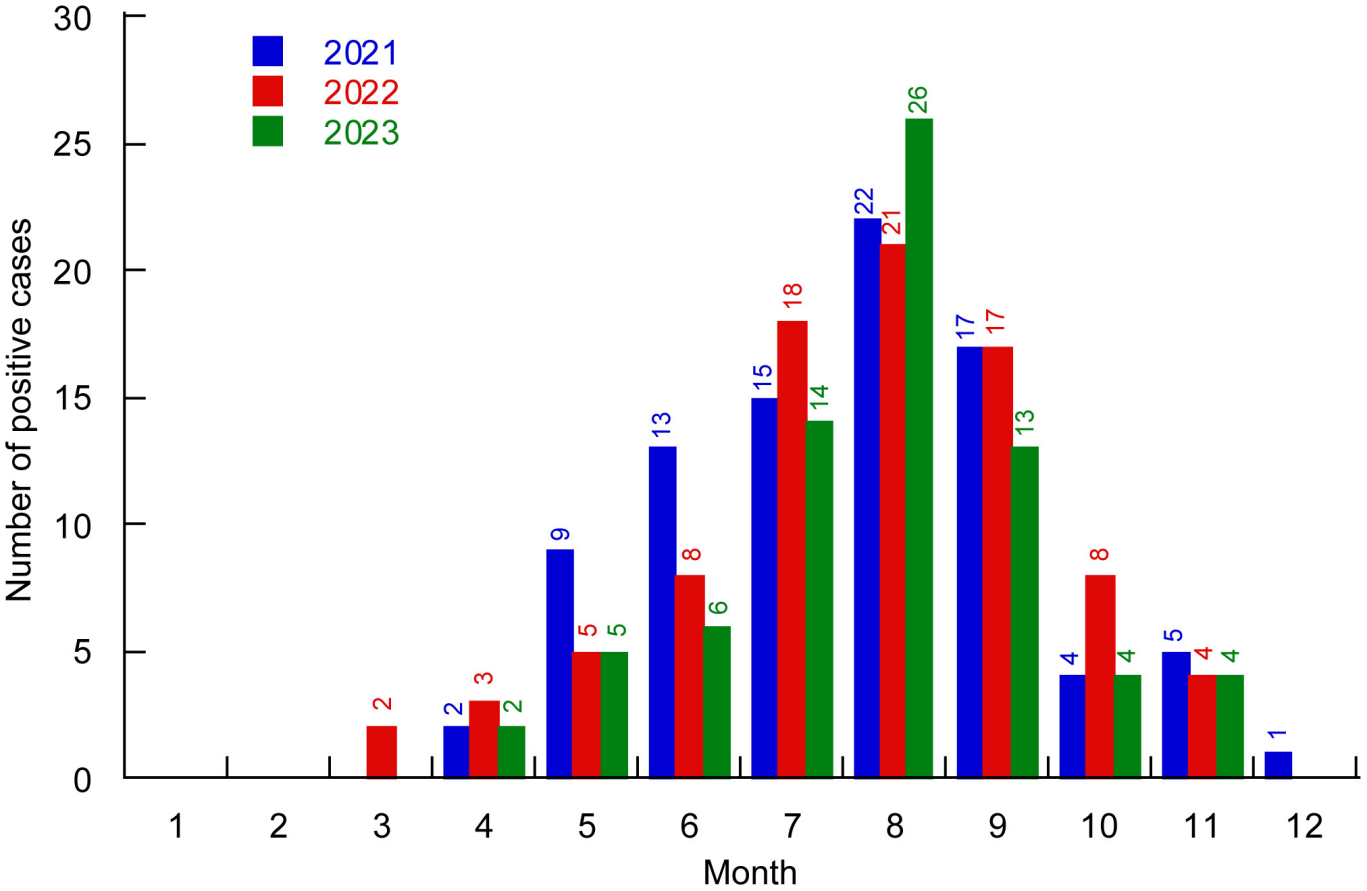
Distribution of *V. parahaemolyticus* positive cases in different months of Huzhou City from 2021 to 2023. Different colors represent different years.

From 2021 to 2023, the detection rate of *V. parahaemolyticus* among foodborne diarrhea patients exhibited a clear seasonal trend. From March to May (spring), 28 positive cases of *V. parahaemolyticus* were identified from a total of 1,463 samples, resulting in a positive rate of 1.91%. From June to August (summer), 143 positive cases were identified from 2,136 samples, yielding a positive rate of 6.69%. From September to November (autumn), 78 out of 1,709 samples tested positive, with a positive rate of 4.56%. From December to February (winter), only 1 out of 1,096 samples tested positive, with the lowest positive rate of 0.091%. Positive cases were fewer during the winter and spring seasons, peaking in summer and autumn, especially from July to September (Figure 1).

### 3.4 Serovar distribution

Among the 250 *V. parahaemolyticus* strains, 9 O serogroups and 28 serotypes were identified. The most prevalent serovars were O10:K4, constituting 60.00% of the strains (150 out of 250), followed by O3:K6 at 13.20% (33 out of 250), and O4:Kut at 11.20% (28 out of 250). A small fraction, 1.60% (4 out of 250), could not be typed.

In 2021, the strains were distributed across 6 O serogroups and 11 serotypes, with O10:K4 being the most dominant at 81.82% (72 out of 88), followed by O3:K6 at 5.69% (5 out of 88), and O4:Kut at 3.41% (3 out of 88). In 2022, the strains belonged to 5 O serogroups and 12 serotypes, with O10:K4 remaining the most prevalent at 40.70% (35 out of 86), O4:Kut at 27.91% (24 out of 86), and O3:K6 at 13.95% (12 out of 86). In 2023, the strains were categorized into 7 O serogroups and 15 serotypes, with O10:K4 continuing to dominate at 56.68% (43 out of 76), O3:K6 at 21.05% (16 out of 76), and O3:K4 at 3.95% (3 out of 76) (Table 3).

**Table 3:** Serovar distribution of *V. parahaemolyticus* in diarrhea cases in Huzhou city from 2021 to 2023.

**O Serogroup**	**Serotype**	**2021 (*****N*** = **88)**		**2022 (*****N*** = **86)**		**2023 (*****N*** = **76)**		**Summary**	
		**Number**	**Rate (%)**	**Number**	**Rate (%)**	**Number**	**Rate (%)**	**Number**	**Rate (%)**
O1	O1:K17	0	0	0	0	1	1.32	1	0.40
	O1:K36	0	0	0	0	1	1.32	1	0.40
	O1:K56	0	0	0	0	1	1.32	1	0.40
	O1:Kut	0	0	2	2.33	0	0	2	0.80
O2	O2:K59	1	1.14	0	0	0	0	1	0.40
O3	O3:K4	0	0	0	0	3	3.95	3	1.20
	O3:K6	5	5.69	12	13.95	16	21.05	33	13.20
	O3:K7	0	0	1	1.16	0	0	1	0.40
	O3:K37	0	0	2	2.33	0	0	2	0.80
	O3:Kut	1	1.14	1	1.16	0	0	2	0.80
O4	O4:K8	0	0	/	/	1	1.32	1	0.40
	O4:K55	1	1.14	2	2.33	0	0	3	1.20
	O4:K61	0	0	0	0	1	1.32	1	0.40
	O4:K63	1	1.14	0	0	0	0	1	0.40
	O4:Kut	3	3.41	24	27.91	1	1.32	28	11.20
O5	O5:Kut	1	1.14	0	0	0	0	1	0.40
O8	O8:K4	0	0	1	1.16	0	0	1	0.40
	O8:K18	0	0	0	0	1	1.32	1	0.40
	O8:K21	0	0	1	1.16	0	0	1	0.40
	O8:Kut	0	0	3	3.49	0	0	3	1.20
O9	O9:K1	0	0	0	0	1	1.32	1	0.40
O10	O10:K4	72	81.82	35	40.70	43	56.58	150	60.00
	O10:K17	0	0	1	1.16	0	0	1	0.40
	O10:K60	1	1.14	0	0	0	0	1	0.40
	O10:K64	0	0	0	0	1	1.32	1	0.40
	O10:Kut	0	0	0	0	1	1.32	1	0.40
O11	O11:K5	1	1.14	0	0	0	0	1	0.40
	O11:Kut	1	1.14	0	0	1	1.32	2	0.80
Untypable		0	0	1	1.16	3	3.95	4	1.60
Summary		88	100.0	86	100.0	76	100.1	250	100.0

### 3.5 Virulence gene detection results

All 250 strains of *V. parahaemolyticus* were identified as carrying the species-specific gene *tlh*. The most common virulence gene profile among these strains was *tdh*+, representing 93.20% of the total; *trh*+ was found in 4.00% of the strains; and the co-presence of both *tdh*+ and *trh*+ was observed in 1.20% (Table 4). We identified four isolates that carried only the *tlh* gene. *tdh*+ strains were predominantly found in serotypes O3:K6, O10:K4, and O4:KUT, whereas *trh*+ and *tdh*+*trh*+ strains were less prevalent among these three major serotypes. *trh*+ strains were distributed across multiple serotypes, including O1:K17, O1:KUT, O2:K59, O4:K63, O8:KUT, O10:K4, O10:K60, O11:K5, and O11:KUT. In contrast, *tdh*+*trh*+ strains were only detected in O1:KUT, O5:KUT, and O11:KUT.

**Table 4:** Virulence gene profiles across *V. parahaemolyticus* serotypes in Huzhou from 2021 to 2023.

**O serogroup**	**Serovar**	**Genotype**						**Summary**	
		***tdh^+^trh^−^***	**Proportion (%)**	***tdh^−^trh^+^***	**Proportion (%)**	***tdh^+^trh^+^***	**Proportion (%)**	**Number**	**Proportion (%)**
O1	O1:K17	0	0	1	0.40	0	0	1	0.40
	O1:K36	1	0.40	0	0	0	0	1	0.40
	O1:K56	1	0.40	0	0	0	0	1	0.40
	O1:Kut	0	0	1	0.40	1	0.40	2	0.80
O2	O2:K59	0	0	1	0.40	0	0	1	0.40
O3	O3:K4	2	0.80	0	0	0	0	2	0.80
	O3:K6	33	13.20	0	0	0	0	33	13.20
	O3:K7	0	0	0	0	0	0	0	0
	O3:K37	1	0.40	0	0	0	0	0	0.40
	O3:Kut	2	0.80	0	0	0	0	2	0.80
O4	O4:K8	1	0.40	0	0	0	0	1	0.40
	O4:K55	3	1.20	0	0	0	0	3	1.20
	O4:K61	1	0.40	0	0	0	0	1	0.40
	O4:K63	0	0	1	0.40	0	0	1	0.40
	O4:Kut	28	11.20	0	0	0	0	28	11.20
O5	O5:Kut	0	0	0	0	1	0.40	1	0.40
O8	O8:K4	1	0.40	0	0	0	0	1	0.40
	O8:K18	1	0.40	0	0	0	0	1	0.40
	O8:K21	1	0.40	0	0	0	0	1	0.40
	O8:Kut	2	0.80	1	0.40	0	0	3	1.20
O9	O9:K1	1	0.40	0	0	0	0	1	0.40
O10	O10:K4	149	59.60	1	0.40	0	0	150	60.00
	O10:K17	0	0	0	0	0	0	0	0
	O10:K60	0	0	1	0.40	0	0	1	0.40
	O10:K64	1	0.40	0	0	0	0	1	0.40
	O10:Kut	1	0.40	0	0	0	0	1	0.40
O11	O11:K5	0	0	1	0.40	0	0	1	0.40
	O11:Kut	0	0	1	0.40	1	0.40	2	0.80
Untypeable		3	1.20	1	0	0	0	4	1.60
Summary		233	93.2	10	4.00	3	1.20	246	98.40

### 3.6 Susceptibility test results

A random selection of 180 *V. parahaemolyticus* strains were subjected to susceptibility testing. The resistance rate to CFZ was as high as 95.56% (172 out of 180). The resistance rates for other antibiotics were: AMP at 12.78% (23 out of 180), NAL at 3.89% (7 out of 180), AMS and TET at 3.33% (6 out of 180), and CTX and CAZ at 1.11% (2 out of 180). In contrast, all strains were 100.0% sensitive to CFX, IPM, GEN, CHL, CIP, and SXT (Table 5).

**Table 5:** Antibiotic resistance of *V. parahaemolyticus* in Huzhou from 2021 to 2023.

**Categories**	**Antibiotics**	**R**	**I**	**S**
		Number/Rate (%)	Number/Rate (%)	Number/Rate (%)
Penicillins	AMP	23/12.78	56/31.11	101/56.11
	AMS	6/3.33	2/1.11	172/95.56
Cephalosporins	CTX	2/1.11	2/1.11	176/97.78
	CAZ	2/1.11	0/0.00	178/98.89
	CFX	0/0.00	0/0.00	180/100.00
	CFZ	172/95.56	8/4.44	0/0.00
Carbapenems	IPM	0/0.00	0/0.00	180/100.00
Aminoglycosides	GEN	0/0.00	0/0.00	180/100.00
Tetracyclines	TET	6/3.33	0/0.00	174/96.67
Quinolones	NAL	7/3.89	0/0.00	173/96.11
	CIP	0/0.00	0/0.00	180/100.00
Chloramphenicol	CHL	0/0.00	0/0.00	180/100.00
Sulfonamides	SXT	0/0.00	0/0.00	180/100.00

AMP, Ampicillin; AMS, Ampicillin/sulbactam; CTX, Cefotaxime; CAZ, Ceftazidime; CFX, Cefuroxime; CFZ, Cefazolin; IPM, Imipenem; GEN, Gentamicin; TET, Tetracycline; NAL, Nalidixic acid; CIP, Ciprofloxacin; CHL, Chloramphenicol; SXT, Trimethoprim/sulfamethoxazole. R, Resistant; I, Intermediate; S: Susceptible.

### 3.7 Minimum spanning tree analysis

**Figure 2:**
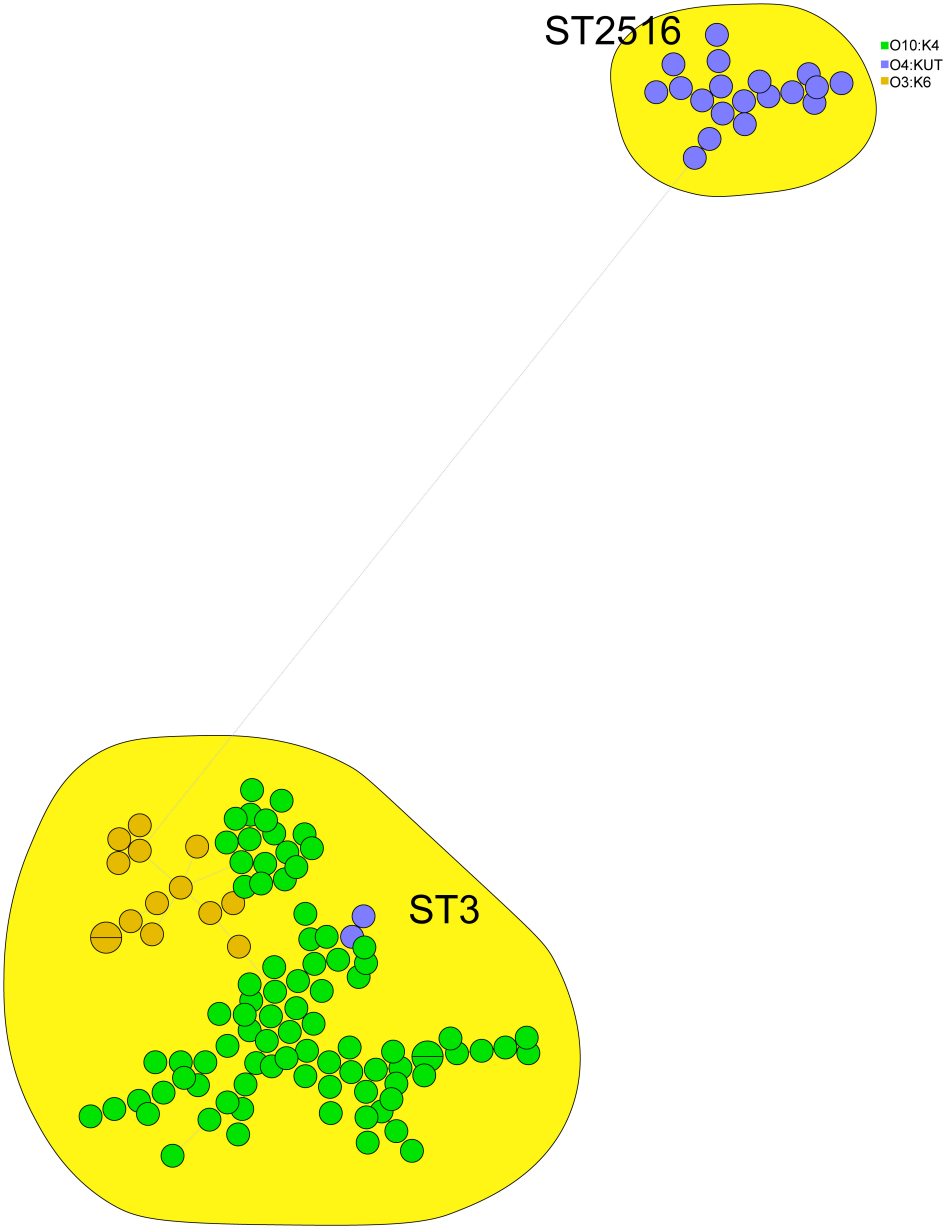
cgMLST based minimum spanning tree of 121 clinical *V. parahaemolyticus* isolates. Circle colors denote different serotypes (O3:K6, O4:KUT, O10:K4), while circle sizes reflect the number of isolates in each node.

We performed cgMLST analysis on 121 clinical isolates, comprising 85 O10:K4, 15 O3:K6, and 21 O4:KUT strains, and constructed a minimum spanning tree. The results demonstrated that these isolates clustered into two major ST types (ST3 and ST2516) with significant genetic divergence. The O4:KUT serotype was distributed across both ST types. Interestingly, the O10:K4 serotype also separated into two distinct clusters, potentially evolving from O3:K6 strains (Figure 2).

## 4 Discussion

This study analyzed the infection status of *V. parahaemolyticus* in foodborne diarrhea cases in Huzhou City from January 2021 to December 2023, with a positive detection rate of 3.90% among 6,404 diarrhea cases. The detection rate is higher than the average found in Mainland China (2.08%) (Wang et al., 2021), likely attributed to Huzhou's geographic location and dietary customs. Huzhou City is rich in aquatic products, and its residents have a tradition of consuming raw or undercooked aquatic products. With the development of logistics, various live seafood products are increasingly sold in inland areas. According to the report, the contamination rate of *V. parahaemolyticus* in inland provinces has reached 23.14% (Pang et al., 2020). Therefore, there is a possibility of cross-contamination between seafood and freshwater products during sales and processing (Chen et al., 2021; Liao et al., 2015), leading to infections when people consume contaminated aquatic products.

The temporal distribution shows that *V. parahaemolyticus* infections can occur throughout the year except for January and February, with the majority of cases concentrated in July to September (Huang et al., 2023), which is consistent with the characteristic of high incidence in summer and autumn. According to Zhejiang Meteorological Bureau or Huzhou Meteorological Station reports, from 2021 to 2023, Huzhou experienced significant climatic variability driven by the alternating influences of El Niño-Southern Oscillation (ENSO) phenomena. The extended La Niña conditions (2020–2022) resulted in distinct seasonal patterns, characterized by colder-than-average winter temperatures and increased precipitation variability. Conversely, the emerging El Niño phase in 2023 contributed to intensified summer heatwaves and altered precipitation regimes, consistent with broader warming trends observed across eastern China (https://epmap.zjol.com.cn/jsb0523/202303/t20230310_25510472.shtml). High temperatures during these months facilitate bacterial growth and reproduction (Neil et al., 2023; Cantet et al., 2013; Fernandez-Velez et al., 2023). Our study has shown that sporadic diarrhea cases are mainly concentrated in middle-aged and young people (aged 25 to < 65 years), which was consistent with previous research (Wang et al., 2021), possibly because this age group has more opportunities to dine out compared to other age groups, and the abundance of night markets in summer and autumn increases the risk of infection when consuming undercooked or raw aquatic products (Li et al., 2020). Consistent with previous findings, the patients aged ≥65 years were the least likely to get infectious diarrhea (Gong et al., 2018). This diversity of age distribution might reflect a natural change in host immunity (Simon et al., 2015) and/or dietary habits that are related to age (Jiang et al., 2024). These findings of the effect of age on the pathogen detection might provide scientific evidence for finding the optimal timing to enhance prevention measures for *V. parahaemolyticus*.

*V. parahaemolyticus* produces thermolabile hemolysin (TLH), thermotolerant direct hemolysin (TDH), and thermotolerant direct hemolysin-related hemolysin (TRH), encoded by the *tlh/tdh/trh* genes are considered to be key indicators of *V. parahaemolyticus* pathogenicity (Lee et al., 2019; Broberg et al., 2011; Gutierrez West et al., 2013; Shirai et al., 1990). TDH and TRH are considered the most critical virulence factors of *V. parahaemolyticus* (Ceccarelli et al., 2013), with most clinical isolates producing one or both of these hemolysins (Letchumanan et al., 2014). The results of this study show that the positive rate of the *tdh* virulence gene is 93.20%, the *trh* virulence gene is 4.00% and the simultaneous positivity of *tdh* and *trh* virulence genes is 1.20% which was consistent with other study (Sun et al., 2022). Several studies have also reported that around 2.0% of the clinical strains do not contain *tdh* and/or *trh* (Li et al., 2014; Pazhani et al., 2014). Even in the absence of these hemolysins, *V. parahaemolyticus* remains pathogenic indicating that other virulence factors exist (Jones et al., 2012; Mahoney et al., 2010). It was reported that beyond hemolysins, the virulence mechanisms of *V. parahaemolyticus* critically depend on specialized secretion systems that mediate effector delivery into host cells (Orlova, 2015). Notably, the bacterium employs two functionally distinct type III secretion systems (T3SS): T3SS1 facilitates host cell death through the translocation of cytotoxic effectors (VopQ, VopS, VPA0450, and VopR/VP1638), while T3SS2 secretes a distinct repertoire of effectors (VopC, VopT, VopZ, VopA/P, VopV, VopL, and VPA1380) that collectively suppress host cell proliferation and induce cytotoxicity (Chatterjee et al., 2013). Additionally, the type VI secretion system (T6SS) contributes to pathogenicity through dual functionality—delivering virulence effectors not only into eukaryotic host cells but also competing bacterial cells (Sha et al., 2013; Li et al., 2019).

In 1996, the O3:K6 serotype of *V. parahaemolyticus* caused a large-scale food poisoning outbreak in India and subsequently became pandemic globally. O3:K6, along with 21 other serotypes, is known as the “pandemic clone” of *V. parahaemolyticus* and is the most frequently reported serotype worldwide (Okuda et al., 1997). Before 2021, the O3:K6 serotype was the main serotype in foodborne diarrhea cases in this region (Zhang et al., 2022). The results of this study show that from 2021 to 2023, the O10:K4 serotype accounted for 60.00% of foodborne diarrhea cases in Huzhou across all seasons, replacing O3:K6 as the dominant serotype in the region. In fact, in 2020, O10:K4 serotype was detected for the first time in Huzhou City. Afterwards, O10:K4 serotype surpassed O3:K6 as the new dominant serotype (Zhang et al., 2022). In recent years, the O10:K4 serotype has dominated in acute gastroenteritis outbreaks caused by *V. parahaemolyticus* in other provinces in China (Wu et al., 2024; Huang et al., 2022a,b). The emergence of serotype O10:K4 may be the response to host immunologic pressure (Huang et al., 2022a). A previous study indicates that O10:K4 strains and the genetic variant O3:K6 were placed in the same cluster, suggesting a possibility of transfer of the pathogenic genes (Huang et al., 2022b).

With the widespread and extensive use of antibiotics in clinical and breeding fields, the issue of bacterial resistance is becoming increasingly severe. The results of this study show that the resistance rate of *V. parahaemolyticus* to CFZ in Huzhou from 2021 to 2023 was as high as 95.56%; some strains exhibited resistance to AMS, CTX, CAZ, TET, and NAL. The resistance rate of local *V. parahaemolyticus* to CFZ was similar to that of clinical isolates from Nantong (99.1%) and from isolates from Nanjing (99.2%) (Zhou et al., 2022; Huang et al., 2023). However, it was significantly higher than the resistance rate reported for domestic clinical isolates a decade ago (50.4%) (Chen et al., 2016). A previous research has indicated that *V. parahaemolyticus* exhibits a notably high resistance to AMP in recent years (Dahanayake et al., 2020; Lopatek et al., 2018; Mok et al., 2021; Nishino et al., 2021; Vu et al., 2022). However, our study reveals that the organism demonstrates the highest resistance to CFZ, with resistance to AMP at a comparatively lower rate of 12.78%, aligning with previous findings (Zhang et al., 2024a), which may be related to the types of antibiotics used in aquaculture or clinical practice, leading to a decrease in ampicillin resistance. Currently, tetracycline, cephalothin, and quinolone drugs are considered first-line options for the treatment of *V. parahaemolyticus* infections (Elmahdi et al., 2016; Han et al., 2007; Tan et al., 2017). Earlier study reported 100% sensitivity of *V. parahaemolyticus* to the AMS (da Silva et al., 2021), our findings show a concerning development, with 3.33% of strains exhibiting resistance to this antibiotic regimen. The results of this study indicate that, apart from penicillins and cephalosporins, other antibiotics can be used as first-line treatments for *V. parahaemolyticus*. Therefore, continuous resistance monitoring of *V. parahaemolyticus* in foodborne diarrhea cases can timely grasp the trend of resistance changes, which is helpful for guiding rational clinical medication.

In this study, we identified two sequence types (ST3 and ST2516) among 121 clinical *V. parahaemolyticus* isolates (Figure 2). MLST analysis revealed that serotypes O10:K4, O3:K6 and O4:KUT belonged to these two STs, with O4:KUT strains distributed across both ST types and showing significant genetic divergence—a finding consistent with previous reports (Zhang et al., 2024b; Huang et al., 2022a). ST3 emerged as the predominant clinical type, aligning with prior studies that established its clinical significance as an epidemic clone circulating in Asia and America (Chen et al., 2016; Turner et al., 2013). These observations collectively demonstrate the crucial role of ST3 in human infections. Additionally, the O4:KUT strains of ST2516 warrant further investigation due to their potential clinical importance.

In summary, our analysis of *V. parahaemolyticus* infections across six hospitals in Huzhou (2021–2023) revealed that most cases occurred among middle-aged and young adults, with a distinct seasonal concentration during summer months. The predominant serotypes among clinical isolates were O10:K4, O3:K6, and O4:KUT. CgMLST analysis demonstrated significant phylogenetic divergence between some O4:KUT strains and the O10:K4/O3:K6 clusters.

## Data Availability

The original contributions presented in the study are included in the article/Supplementary material, further inquiries can be directed to the corresponding author.
